# Liposarcoma of the spermatic cord mimicking a left inguinal hernia: a case report and literature review

**DOI:** 10.1186/1477-7819-11-18

**Published:** 2013-01-25

**Authors:** Fubiao Li, Runhui Tian, Changjiu Yin, Xiaofan Dai, Hongliang Wang, Ning Xu, Kaimin Guo

**Affiliations:** 1 Department of Andrology, First Hospital of Jilin University, Changchun, Jilin, China; 2 Department of Psychology, First Hospital of Jilin University, Changchun, Jilin, China; 3 Department of Anesthesiology, Women’s and Children’s Hospital of Jilin Province, Changchun, Jilin, China; 4 Department of Urology, First Hospital of Jilin University, Changchun, Jilin, China

**Keywords:** Liposarcoma, Spermatic cord, Inguinal hernia, Literature review

## Abstract

Liposarcoma of the spermatic cord (LSC) is a rare condition characterized by a painless inguinal or scrotal mass. To our knowledge, only about 200 cases have been previously reported in the literature. These tumors are often mistaken for common scrotal swellings, such as hydroceles and hernias. We present a LSC case in which a definitive diagnosis was obtained upon histological examination. We also provide a literature review of other cases that have been reported.

## Background

Liposarcoma is a rare soft tissue malignancy with aggressive behavior and poor prognosis. It is derived from mesenchymal tissue and can occur in fat cells anywhere in the body. Most malignant paratesticular tumors are sarcomas but 5 to 7% are liposarcomas [1]. To the best of our knowledge, only about 200 cases of liposarcoma of the spermatic cord (LSC) have previously been reported worldwide [1-29]. Most were reported in adults, presenting as a painless inguinal or scrotal mass, and were usually mistaken for an inguinal hernia or testicular hydrocele. Preoperative diagnosis was infrequent. Hence, increasing the understanding of LSCs is particularly important. Until now, the published literature on LSC has been limited to case reports with limited clinical information. Here we present a case report and a comprehensive literature review with the objective of providing useful information on this malignancy.

## Case presentation

A 53-year-old male presented with a slow-growing, painless, left scrotum mass of two years duration and was admitted to the outpatient general surgery department for hernioplasty in December 2010. The provisional diagnosis made by his family practitioner was a left–sided inguinal hernia, and the patient was referred for surgery. Physical examination showed a large, non-tender, mobile left scrotal mass. The mass was larger with increased abdominal pressure when the patient was standing, but smaller when he was supine. Trans-illumination testing was negative. There were no constitutional symptoms, voiding complaints, history of local trauma, infection, weight loss or hereditary disease. All pre-operative laboratory tests, including complete blood count, biochemistry and chest X-ray, were normal. A pelvic computerized tomography (CT) scan was negative for retroperitoneal metastasis. Scrotal ultrasonography (8 to 12 linear array transducer, LOGIQ P5, GE Healthcare, New York,New York State,USA) revealed a 55 x 42 mm mass on the left side of the inguinal canal with internal echogenecity resembling fatty tissues and extending to the scrotum.

The patient underwent exploratory surgery via a left inguinal canal approach, during which a well-defined 6 × 5 × 3 cm round mass located above the left testis and epididymis was discovered; the vas deferens was involved. Upon close inspection, there was no evidence of hernia or laxity in the inguinal floor. Intraoperative frozen-section biopsy showed malignancy of the spermatic cord. A complete radical left orchidectomy was performed with wide excision and high ligation of the spermatic cord. An ipsilateral inguinal lymph node was also removed for biopsy.

**Figure 1:**
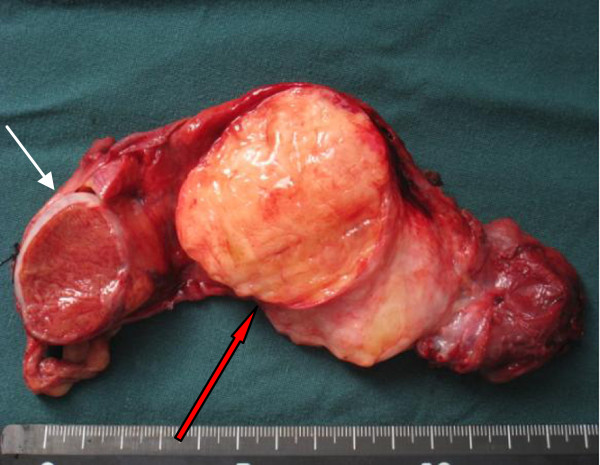
**The gross appearance of LSC. **Macroscopic appearance of the surgical specimen showing an encapsulated mass of adipose tissue and a cut surface having a yellowish lipoma-like texture. The left testis was not infiltrated. (white arrow: normal testis; red blank arrow: tumor tissue).

The gross appearance was a solid mass of adipose tissue with a yellowish lipoma-like texture of the cut-surface. It was encapsulated, and attached to the spermatic cord. A hard tumor could be palpated in the center of the mass (Figure 1).

**Figure 2:**
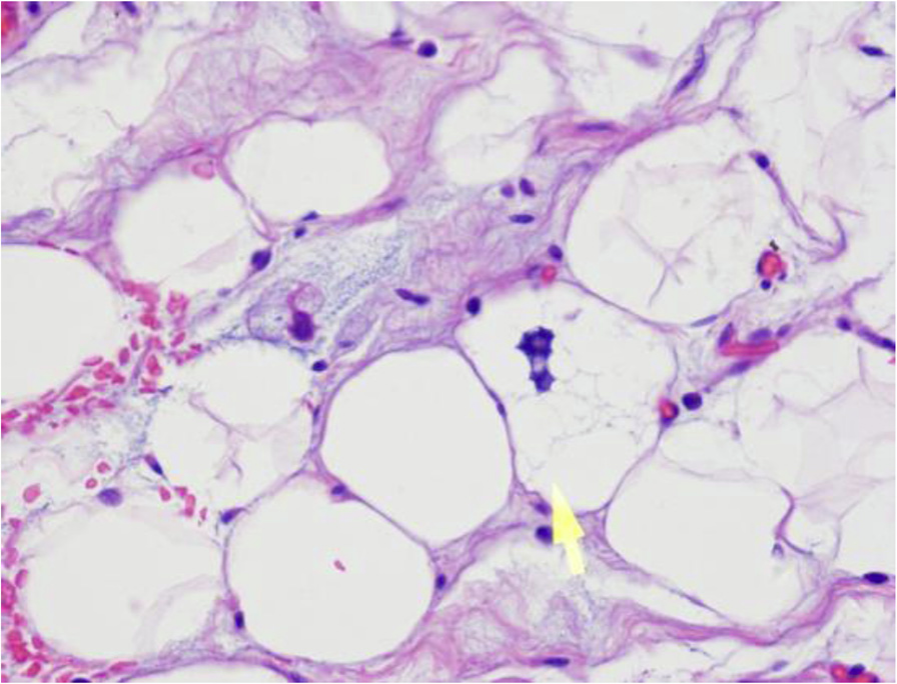
**HE staining of LSC. **Microscopically well-differentiated myxoid liposarcoma composed of mature adipose tissue and a few scattered lipoblasts (yellow arrow: mature fatty cells) (H&E ×400).

Histological examination confirmed a well-differentiated myxoid liposarcoma, composed of mature adipose tissue and a few scattered lipoblasts separated by fibrous septa into the lobules of varying sizes. Lipoblasts with hyperchromatic nuclei, irregularly-shaped spindle cells and abnormal cells were present in myxoid areas (Figure 2). The surgical margin was free of tumor. The left inguinal sentinel lymph node biopsy showed no evidence of metastasis. The patient had a good postoperative clinical course without complications and was discharged on the seventh postoperative day. After an 18-month follow-up without adjuvant therapy, the patient was in good condition with no evidence of recurrence. No metastasis was seen during this period.

## Discussion

Liposarcoma of paratesticular tissues (spermatic cord, testicular tunics or epididymis), first reported in 1952 [2], is a rare neoplasm that comprises approximately 5% to 7% of paratesticular sarcomas [3,4]. Most originate in the spermatic cord [5] but some originate in the retroperitoneum and develop in the inguinal region, involving the spermatic cord [6]. Yoshino *et al*. described a patient who developed LSC after radical prostatectomy for prostate cancer [7]. Manzia *et al*. reported a case of a renal transplant recipient in whom LSC presented on the same side as the graft at four years post-transplantation [8]. The tumor occurs world-wide, although there is a remarkably high incidence among Japanese men, who account for one fourth of published cases. For this literature review, we searched relevant case reports published in English that were available in full-text. Some cases that did not contain detailed information on treatment and outcomes were excluded. As a result, a total of 38 cases documented in 29 published papers were included in our review (Table 1).

**Table 1:** Characteristics and clinical course of published cases of liposarcoma of the spermatic cord

**No.**	**age (y)**	**Duration**	**Location**	**Size (cm)**	**Treatment**	**Pathology**	**Follow –up**	**Outcome**
1^2^	53	NA	L.s	6 × 7	RO	WDL	3 yr	Recurrence
2^2^	70	6 mo	R.s	13 × 7 × 3.5	RO	WDL	6 yr	Recurrence
3^2^	34	NA	L.s	1.5 × 2.5	RO	WDL	4 yr	NRD
4^2^	56	10 yr	L.s	8.5 × 7.5 × 4.5	RO+rad	PDL, WDL	18 mo	NRD
5^2^	53	NA	L.s	NA	RO	DL	18 mo	NRD
6^2^	71	2 yr	L.s	NA	RO	WDL	3 mo	NRD
7^5^	59	5 yr	L.s	50 × 27.5 × 15 (10.9 kg)	RO	WDL	3 yr	NRD
8^6^	60	7 mo	R.s; RP	14 × 3.8 × 3.5; 24 × 10 × 8	TR; RO	WDL (St)	4 yr	NRD
9^7^	71	5 mo	L.s	9.5 × 5.5	RO	DL	4 mo	NRD
10^8^	52	3 mo	L.s	0.4	TR	WDL	3 yr	NRD
11^10^	69	1 yr	L.s	16 × 6 × 5	RO+rad	WDL (St)	NA	NA
12^11^	53	6 mo	R.s	7.5 × 4.5 × 4	RO	WDL (St)	NA	NA
13^12^	76	1 wk	L.s	6 × 5 × 3	TR	WDML	NA	NA
14^13^	24	6 mo	R.s	5	TR	WDML	1 yr	NRD
15^14^	79	3 mo	L.s	12 × 6 (730 g)	RO	ML	1 yr	NRD
16^15^	60	4 yr	R.s	15 × 20 (760 g)	RO	WDML	NA	NA
17^16^	44	1 yr	L.s	20 × 15	RO	WDL (St)	1 yr	NRD
18^17^	60	6 yr	R.s	5 × 4	RO	WDL	3 yr	NRD
19^18^	75	18 mo	L.s	20 × 15	RO	ML	NA	NA
20^18^	54	NA	L.s	20 × 20	RO	PL	NA	NA
21^19^	73	6 mo	R.s	15 × 10 × 14	RO	WDL	8 yr	NRD
22^19^	47	NA	R.s	3	RO	WDL	20 mo	NRD
23^20^	73	1 yr	R.s	17 × 13 × 4	RO rad	DL	2 yr	NRD
24^20^	68	1mo	R.s	8 × 6 × 6	RO	WDL (St)	6 mo	NRD
25^22^.	73	NA	L.s; RP	4.1 × 3.5 × 3; 3.3 × 3.3 × 2	RO; TR	NA	6 mo	NRD
26^23^	40	NA	R.s	50 × 50 × 35 (42 kg)	TR	WDL (St)	12 mo	NRD
27^24^	65	NA	R.s	34 × 22 × 17 (5,786 g)	RO	DL	48 mo	NRD
28^25^	75	NA	NA	14 × 8 × 9	RO	PL	NA	NA
29^26^	47	6 mo	L.s	4 × 3 × 3; 4 × 2 × 2	RO	WDML; A	30 mo	NRD
30^27^	73	18 mo	L.s	10 × 8 × 7	RO	ML	NA	NA
31^29^	65	1 yr	R.s	14 × 8 × 5	RO	WDML	3 mo	NRD
32^30^	64	4 yr	L.s	26 × 15 × 7 (785 g)	RO	WDL	3 mo	NRD
33^32^	57	1.5 yr	L.s	11 × 7.5 × 5	RO+rad	DL	10 yr	NRD
34^32^	60	NA	R.s	3 × 1.8 × 1.5	RO	WDL (St)	1 yr	Recurrence
35^33^	57	1 yr	L.s	9 × 6.5	RO+rad	DL	NA	NA
36^34^	66	3 mo	R.s	5 × 10	RO	PDML	6 mo	Metastases
37^35^	60	1 yr	L.s	10 × 10 × 5	RO	WDL (St)	3 yr	Recurrence
38^36^	48	2 yr	R.s	NA	RO	WDL	3 yr	NRD
Our case	53	2 yr	L.s	6 × 5 × 3	RO	WDML	18 mo	NRD

Abbreviations: A, angiolipoma; DL, dedifferentiated liposarcoma; L.s, left side; ML*, *myxoid liposarcoma; NA*, *not available; NRD*, *no recurrence of disease; PDL*, *poorly-differentiated liposarcoma; PDML*,* poorly-differentiated myxoid liposarcoma; PL*, *pleomorphic liposarcoma; rad*, *radiotherapy; R.s, right side; RO, radical orchiectomy; RP, retroperitoneum; St*, *sclerosing type; TR, tumor resection; WDL*,* well-differentiated liposarcoma; WDML*, *well-differentiated myxoid liposarcoma.

The tumor occurred more frequently in adults than children, with a range of 24 to 79 years of age and a mean age at presentation of 61 years. Overall, 22 of 38 cases (57.9%) were 60 years of age or older. The duration of disease ranged from one week to five years. The typical clinical manifestation of LSC was a slowly growing, non-tender, painless, nodular mass of varying size, located intra-scrotally above the testis or in the groin [9]. Only a few cases presented with a painful node [5,10-12], preoperative diagnosis was not common and was often confused with an inguinal hernia, hydrocele or spermatocele, or a tumor of the testis or epididymis [2,6,10,13-19]. In the present case, the scrotal mass was palpable when the patient was in the upright position and disappeared when he was lying down. It was thus easily mistaken for an inguinal hernia.

High-resolution ultrasonography, computed tomography with contrast and magnetic resonance imaging (MRI) have become the imaging modalities of choice for the examination of the scrotum and its contents, and all can provide useful information about the lipomatous nature of these masses [7,12,20]. Ultrasonography typically reveals a solid, hyperechoic, heterogenous lesion separate from the testicle and similar to benign lipomas. CT usually demonstrates a mass with fat attenuation intermixed with non-lipomatous septa or soft tissue nodules [21].

Vorstman *et al.* found that the neoplasms were dominant on the right side [9]. However, in our literature review, we found that more cases occurred on the left [21] than on the right side [22]. Furthermore, there were three cases in which the retroperitoneum was involved [2,6,23], which strongly suggested that preoperative pelvic CT scanning was necessary to rule out the possibility of a tumor. The tumor size ranged from 0.4 cm to 50 cm with a mean size of 12.5 cm. The tumor weights varied from less than a gram to 42 kg [8,24]. Giant LSC was reported in three cases [24-26].

Grossly, liposarcoma resembles lipoma, especially the lipoblastic types, but the surface may show foci with a mucinus appearance. In large tumors, multinodularity and multilocularity of fatty or cartilaginous tissue were often observed [12,25,27]. Histologically, liposarcomas were divided into well differentiated, dedifferentiated (high and low grade) and myxoid/round cell. Most LSC were low grade, well-differentiated tumors. In the literature we reviewed, well-differentiated liposarcoma (WDL) and myxoid liposarcoma (ML) were the most commonly encountered types, accounting for 48.7% (19/39) and 25.6% (10/39), respectively. Of 19 cases with WDL, 7 patients had a sclerosing subtype, 11 did not report a specific subtype, and only 1 presented with a mixture of WDL and PDL. Dedifferentiated liposarcoma (DL) and (pleomorphic liposarcoma) PL were considered to be highly malignant, and had an incidence of 17.9% (7/39) and 5.1% (2/39), respectively. Some uncommon histological findings have been reported. Domşa described a mixed type liposarcoma with well differentiated major pleomorphic and minor sclerosing components, [26]. Ikinger *et al*. reported a case of a well-differentiated myxoid liposarcoma (WDML) combined with angiolipoma [27]. Although immunohistochemical markers were applied in several cases [13,21,26], accurate diagnoses depended on morphological criteria.

Liposarcomas tend to spread primarily by local extension. Once diagnosed or suspected preoperatively, radical orchiectomy with wide local excision and high ligation of the spermatic cord is recommended [28-30], as was performed in our case. Retroperitoneal lymph node dissection is not indicated unless there is evidence of metastasis. The resection must be wide, and scrotectomy may be considered in patients with high-grade tumors to prevent local recurrence [31]. In the 38 cases reviewed, only four patients underwent tumor resection [8,12,13,24]. Two patients with retroperitoneal involvement were managed by tumor resection and radical orchidectomy [6,23]. All liposarcoma types frequently recur and spread by direct invasion. Only one patient underwent multiple organ resection for an LSC involving the left colon that obstructed the left ureter with loss of left kidney function [2]. Liposarcoma is relatively radiosensitive and radiotherapy is regarded as useful to prevent local recurrence. However, few data are available regarding the optimum radiation dosage. Radiotherapy is only recommended in selected patients whose pathological findings show intermediate or high histological grade or recurrent form. In our review, only four cases underwent radiotherapy because of DL or PDL in histopathology or invasion of section margins [2,20,32,33]. There is no consensus on the benefit of adjuvant chemotherapy. Given the high rate of local recurrence of LSC (55 to 70%), long-term periodic follow-up is mandatory. We found that recurrence was reported in nine cases, including five that occurred after follow-up [2,32,34,35] and four that occurred before [2,24,32,36]. One case recurred four times after an initial inguinal orchiectomy had been performed [2]. The average delay until recurrence was 3.3 years with a range of 1 to 6 years.

In spite of the likelihood of recurrence, the prognosis was satisfactory and the rate of mortality was reduced if radical orchiectomy resulted in complete clearance with a negative margin. Even if patients underwent incomplete resection, improved disease-free survival could be achieved by re-operative wide resection [28]. Tumor size and absence of metastasis at diagnosis remained significant predictors of disease-specific survival [37].

The one reported death was of a patient with PDML who developed widespread metastases after six months follow-up and underwent chemotherapy [2]. Detailed follow-up data and outcomes for two cases with PL were not available. WDL and ML have relatively better outcomes, and have not recurred with a lower grade or in a well-differentiated form.

## Conclusions

We report a rare variety of spermatic cord mass having a misleading presentation. LSC is a very rare condition that can be encountered in urology or outpatient general surgery departments. It should be highly suspected in patients experiencing recurrent hernias of the inguinal region. Therefore, all surgeons should be aware of this malignancy. Careful clinical and radiological examination is helpful for appropriate preoperative diagnosis. The treatment of choice is radical orchiectomy and wide excision with high ligation of the spermatic cord. If the margin is in doubt, adjuvant radiotherapy is indicated. Given the unfavorable prognosis of sarcomatous tumors and the high frequency of recurrence, long-term periodic follow-up is necessary.

## Consent

Written informed consent was obtained from the patient for publication of this case report and any accompanying images. A copy of the written consent is available for review by the Editor-in-Chief of this journal.

## Abbreviation

A: Angiolipoma; DL: Dedifferentiated liposarcoma; LSC: Liposarcoma of spermatic cord; L.s: Left side; ML: Myxoid liposarcoma; NA: Not available; NRD: No recurrence of disease; PDL: Poorly-differentiated liposarcoma; PDML: Poorly-differentiated Myxoid liposarcoma; PL: Pleomorphic liposarcoma; rad: Radiotherapy; RO: Radical orchiectomy; RP: Retroperitoneum; R.s: Right side; St: Sclerosing type; TR: Tumor Resection; WDL: Well-differentiated liposarcoma; WDML: Well-differentiated myxoid liposarcoma.

## Competing interests

The authors declare that they have no competing interests.

## Authors’ contribution

FL conceived of the study design. RT and CY collected relevant literature and modified the draft. NX and HW performed the operation. KG wrote the initial draft. XD checked the manuscript. All authors read and approved the final manuscript.
